# Trichostatin A reverses the chemoresistance of lung cancer with high IGFBP2 expression through enhancing autophagy

**DOI:** 10.1038/s41598-018-22257-1

**Published:** 2018-03-02

**Authors:** Dongfang Tang, Ruyong Yao, Dandan Zhao, Lin Zhou, Yun Wu, Yang Yang, Yifeng Sun, Liming Lu, Wen Gao

**Affiliations:** 1 Department of Thoracic Surgery, Shanghai Key Laboratory of Clinical Geriatric Medicine, HuaDong Hospital Affiliated with FuDan University, Shanghai, China; 2 Department of Thoracic Surgery, Shanghai Chest Hospital Affiliated with Shanghai Jiaotong University, Shanghai, China; 3 Central Laboratory of Shanghai Chest Hospital Affiliated with Shanghai Jiaotong University, Shanghai, China; 4 Central laboratory of the Affiliated Hospital of Qingdao University, Qingdao, China

## Abstract

Insulin-like growth factor (IGF) signaling plays an important role in tumorigenesis and metastasis. Here, we analyzed insulin-like growth factor (IGF) binding protein-2 (IGFBP2) expression in 81 lung cancer patients and 36 controls consisting of healthy and benign pulmonary lesion participants for comparison, then validated the IGFBP2 expression in additional 84 lung cancer patients, and evaluated the prognostic and chemoresistant significance of IGFBP2 in two cohorts respectively. Next we detected the reversal effect of trichostatin A (TSA) on chemoresistance in cell lines with high IGFBP2 expression. As a result, the mean expression of IGFBP2 in lung cancer patients was significantly higher than that in controls and increased with lung cancer progressed to advanced stage. In addition, high IGFBP2 expression was independently predictive for chemoresistance; over-expressed IGFBP2 enhances cell activity and TSA can reverse the chemoresistance induced by high IGFBP2 expression through enhancing autophagy. Furthermore, multivariate analysis showed that lung cancer patients whose blood IGFBP2 was higher had a poor survival outcome, with a hazard ratio of 8.22 (95%CI 1.78–37.92, P = 0.007) after adjustment for stage, histopathology, EGFR mutation, age, smoking and surgery.

## Introduction

Despite the identification of aberrant signaling pathways in non-small cell lung cancer (NSCLC) has elucidated mechanisms of disease pathogenesis and led to the development of new molecular targeted therapies^1^, lung cancer remains the most common malignant tumor and the major cause of cancer-related deaths worldwide. The overall 5-years survival rate is only 15%^2^. Further characterization of potentially druggable and prognostically relevant pathways might improve risk assessment and therapeutic strategies for NSCLC patients^3^. However, very few serological cytokines that can be used for prognosis and chemoresistance are clinically available.

Insulin-like growth factor (IGF) binding protein-2 (IGFBP2) is a member of the IGFBP family of proteins, which function as carriers of IGF-I and IGF-II in blood and extracellular fluid, and is the second most abundant IGFBP in the circulation (after IGFBP3)^4^. In addition to IGF-binding domains that are common to all IGFBPs, IGFBP2 contains GlyArg-Asp (RGD) and heparin-binding motifs that bind to integrins and extracellular matrix directly and triggers biological actions independent of IGFs^5^. Unlike IGFBP3, IGFBP2 has been shown to promote tumorigenesis, metastasis, cancer stem cell expansion, and tumor angiogenesis^6,7^. Moreover, overexpression of IGFBP2 has been associated with resistance to docetaxel or paclitaxel and anti-hormone therapy in prostate cancer, suggesting that IGFBP2 induced functional changes in cancer cells could play a critical role in the efficacy of anticancer therapy^8^.

Autophagy is an evolutionarily conserved intracellular process, by which cytoplasm is enveloped in a double-membrane vesicle and shuttled to lysosomes for degradation, participating in stress tolerance^9^. Currently, many pathway component specific inhibitors are in development which potently activate autophagy-induced apoptosis. However, John L. Cleveland *et al*. found that targeting autophagy augments the anticancer activity of SAHA (histone deacetylase inhibitor) to overcome *BCR-ABL*-mediated drug resistance^10^. And Ravi K. Amaravadi *et al*. reported that measurements of tumor cell autophagy predict chemoresistance and survival in melanoma^11^; Therefore, Autophagy is likely to be activated ectopically in tumor cells, which confers to chemoresistance^12,13^.

In this study, we intend to illuminate: (a) Evaluate the role of IGFBP2 in prognosis and chemoresistance in lung cancer; (b) Detect the effect of IGFBP2 on cell viability and reveal the therapeutic effect of histone deacetylase inhibitors (HDACi) on cisplatin-resistant cell lines; (c) Evaluate the role of autophagy in reversing chemoresistence.

## Results

### Aberrant expression of IGFBP2 in NSCLC patients

**Figure 1:**
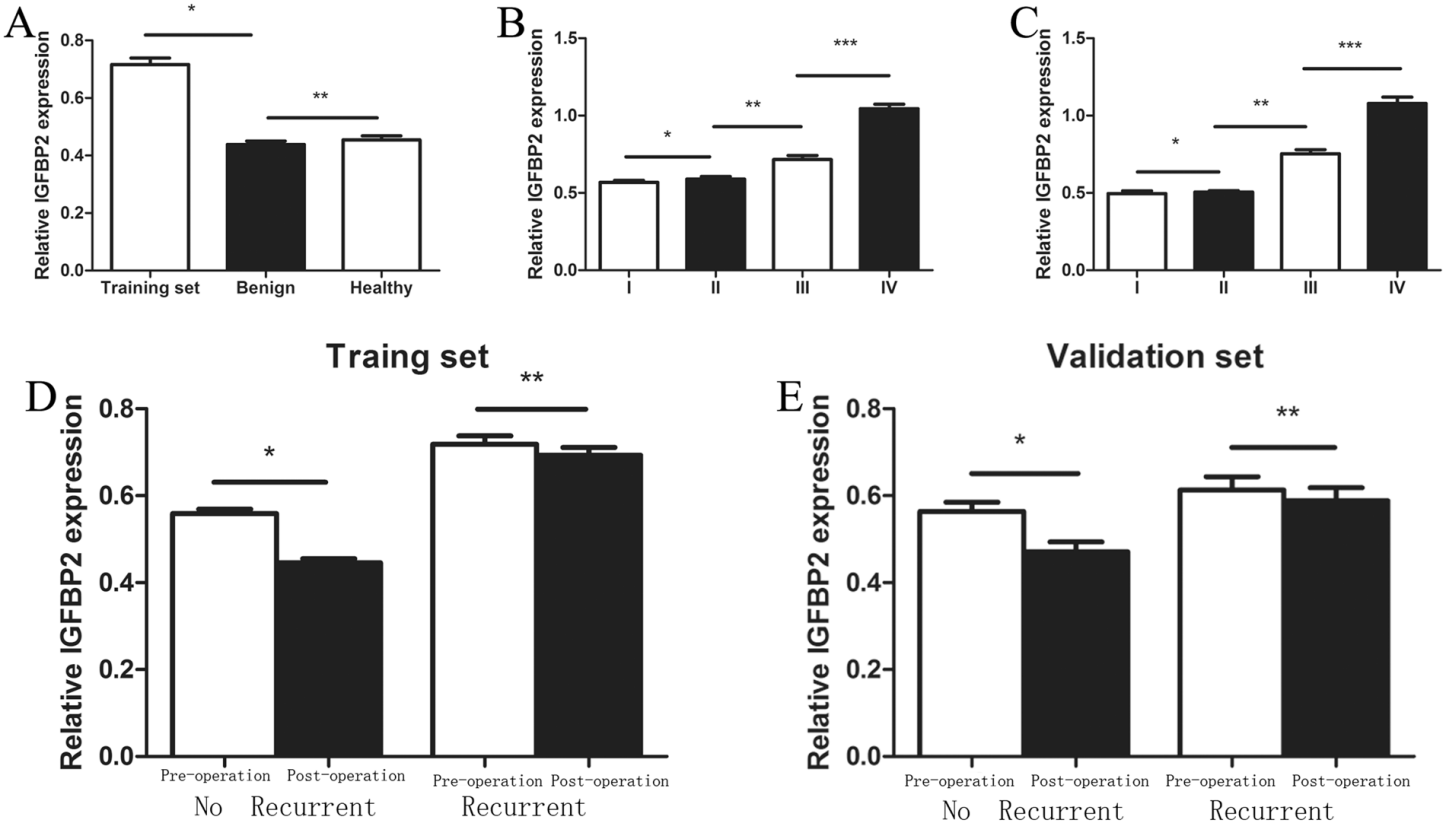
The IGFBP2 expression in training and validation sets. (**A**) The IGFBP2 in lung cancer patients was significantly higher than that in healthy and benign participants. (*represent P < 0.01; **represent P = 0.458). (**B**) IGFBP2 expression increases along with the disease progresses to advanced stage in the training set. (*represent P = 0.300; **represent P = 0.0002; ***represent P < 0.001). (**C**) IGFBP2 expression increases along with the disease progresses to advanced stage in the validation set. (*represent P = 0.596; ** represent P < 0.001; ***represent P < 0.001). (**D**) The expression of IGFBP2 before and after surgery in the training sets, IGFBP2 decreased significantly in patients without early recurrent and did not decrease significantly in patients with early recurrent (*represent P < 0.01; **represent P = 0.400). (**E**) The expression of IGFBP2 before and after surgery in validation sets, IGFBP2 decreased significantly in patients without early recurrent and did not decrease significantly in patients with early recurrent (*represent P = 0.004; **represent P = 0.573).

The characteristics were summarized in Tables 1 and 2. There was no difference in terms of TNM stage, histology, age, gender and smoking history. In order to test whether there is a significant difference in serum IGFBP2 expression level of lung cancer patients, we compared serum samples of 81 lung cancer patients in training set with 36 age-matched healthy and benign participants. When the data were analyzed on the basis of age, gender and smoking history, the results showed that the mean serum concentration of IGFBP2 in lung cancer patients was significantly higher than that in healthy and benign participants (P < 0.001) (Fig. 1A); however, the expressions of IGFBP2 between healthy and benign participants were not significant.

**Table 1:** Patient characteristics and IGFBP2 level in training set.

Variable	Patients(n)	IGFBP2	P-value
Gender			
Male	39	0.731 ± 0.0327	
Female	42	0.702 ± 0.0328	0.527
Age			
<64 years	45	0.751 ± 0.0347	
≥64 years	36	0.673 ± 0.0276	0.094
Smoking			
Never	37	0.692 ± 0.0311	
Ever	44	0.737 ± 0.0335	0.331
Histology			
Adenocarcinoma	41	0.693 ± 0.0315	
Squamous cell carcinoma	40	0.740 ± 0.0338	0.304
Stage			
I	21	0.568 ± 0.0126	
II	22	0.590 ± 0.0162	
III	20	0.715 ± 0.0265	
IV	18	1.044 ± 0.0288	<0.01
EGFR mutation			
Yes	9	0.686 ± 0.0325	
No	32	0.703 ± 0.0626	0.808
Recurrent after Surgery			
Before Surgery	24	0.720 ± 0.0200	
After Surgery	24	0.693 ± 0.0180	0.340
Not recurrent after Surgery			
Before Surgery	37	0.559 ± 0.0100	
After Surgery	37	0.445 ± 0.0095	<0.001
Healthy	24	0.438 ± 0.0120	
Benign lesion	12	0.454 ± 0.0140	0.458

**Table 2:** Patient characteristics and IGFBP2 level in validation set.

Variable	Patients(n)	IGFBP2	P-value
Gender			
Male	43	0.688 ± 0.0403	
Female	41	0.665 ± 0.0357	0.671
Age			
<64 years	41	0.669 ± 0.0343	
≥64 years	43	0.684 ± 0.0414	0.776
Smoking			
Never	36	0.665 ± 0.0492	
Ever	48	0.689 ± 0.0296	0.704
Histology			
Adenocarcinoma	47	0.669 ± 0.0332	
Squamous cell carcinoma	37	0.687 ± 0.0444	0.740
Stage			
I	22	0.496 ± 0.0170	
II	24	0.506 ± 0.0082	
III	22	0.753 ± 0.0259	
IV	16	1.078 ± 0.0420	<0.001
EGFR mutation			
Yes	10	0.655 ± 0.0722	
No	37	0.673 ± 0.0380	0.835
Recurrent after Surgery			
Before Surgery	14	0.613 ± 0.0310	
After Surgery	14	0.588 ± 0.0300	0.573
Not recurrent after Surgery			
Before Surgery	42	0.564 ± 0.0210	
After Surgery	42	0.471 ± 0.0228	0.0037

### IGFBP2 in subgroups of lung cancer patients

The IGFBP2 cutoff value was fixed at 0.65 allowing maximal sensitivity (81.0%) with minimal false-positive rate (35.0%) based on the ROC curve obtained from training set. As a result, the mean IGFBP2 values in the training set were 0.57, 0.59, 0.72, and 1.04 for stage I, II, III, and IV tumors, respectively (Fig. 1B). Average IGFBP2 values were 0.50, 0.51, 0.75, and 1.08 in validation set (Fig. 1C). There was no significant difference between stage I and II in training and validation sets; however, the results were significant when IGFBP2 expressions were compared between stage II and III, stage III and IV and stage I + II and III + IV patients in training and validation sets, suggesting that IGFBP2 increased with disease progressed to advanced stage.

To detect whether IGFBP2 expression level changed with surgery, we also measured the IGFBP2 expression before and post operation in training and validation sets. As a result, the levels of IGFBP2 decreased significantly after surgery in patients without early recurrent in both training and validation sets. For those patients with early recurrent after curative surgery, the levels of IGFBP2 did not decrease significantly (Fig. 1D and E).

Nevertheless, for those adeocarcinoma patients, we analyzed the association between IGFBP2 with EGFR mutation. As a result, there was no significant difference in training and validation sets. There was a higher trend for smoking or ever smoking patients than never smoking patients, however, the difference did not reach significant.

### IGFBP2 expression and chemoresistance in NSCLC patients

**Figure 2:**
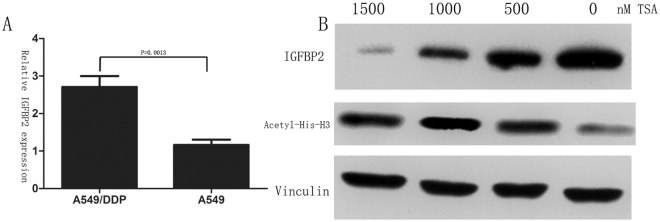
IGFBP2 expression is higher significantly in A549/DDP than A549 cell lines; treatment of A549/DDP with TSA significantly decreases IGFBP2 expression in a dose-dependent targeting to Acetyl-Histone-H3.

High IGFBP2 expression was significantly associated with a higher incidence of primary resistant disease including stable disease (SD) and progressive disease (PD) (IGFBP2 high 88.0%, IGFBP2 low 12.0%; P = 0.0138) and with a lower responding rate including complete response (CR) and partial response (PR) (IGFBP2 high 32.6%, IGFBP2 low 67.4%; P < 0.001). In multivariate analyses, high IGFBP2 expression was independently predictive for chemoresistance (OR = 5.502 (95% CI 1.21–25.12); P = 0.028). Furthermore, we found that cisplatin-resistant A549/DDP cell lines showed higher IGFBP2 expression than A549 cell lines(Fig. 2A); treatment of A549/DDP with Trichostatin A (TSA) significantly decreases IGFBP2 expression in a dose-dependent and acts on Acetyl-Histone-H3 since that we use an antibody that recognizes only an acetylated form of Histone H3 (Fig. 2B), suggesting TSA regulates the IGFBP2 expression in transcription level.

### IGFBP2 enhance the cell viability and TSA reverse the chemoresistance

**Figure 3:**
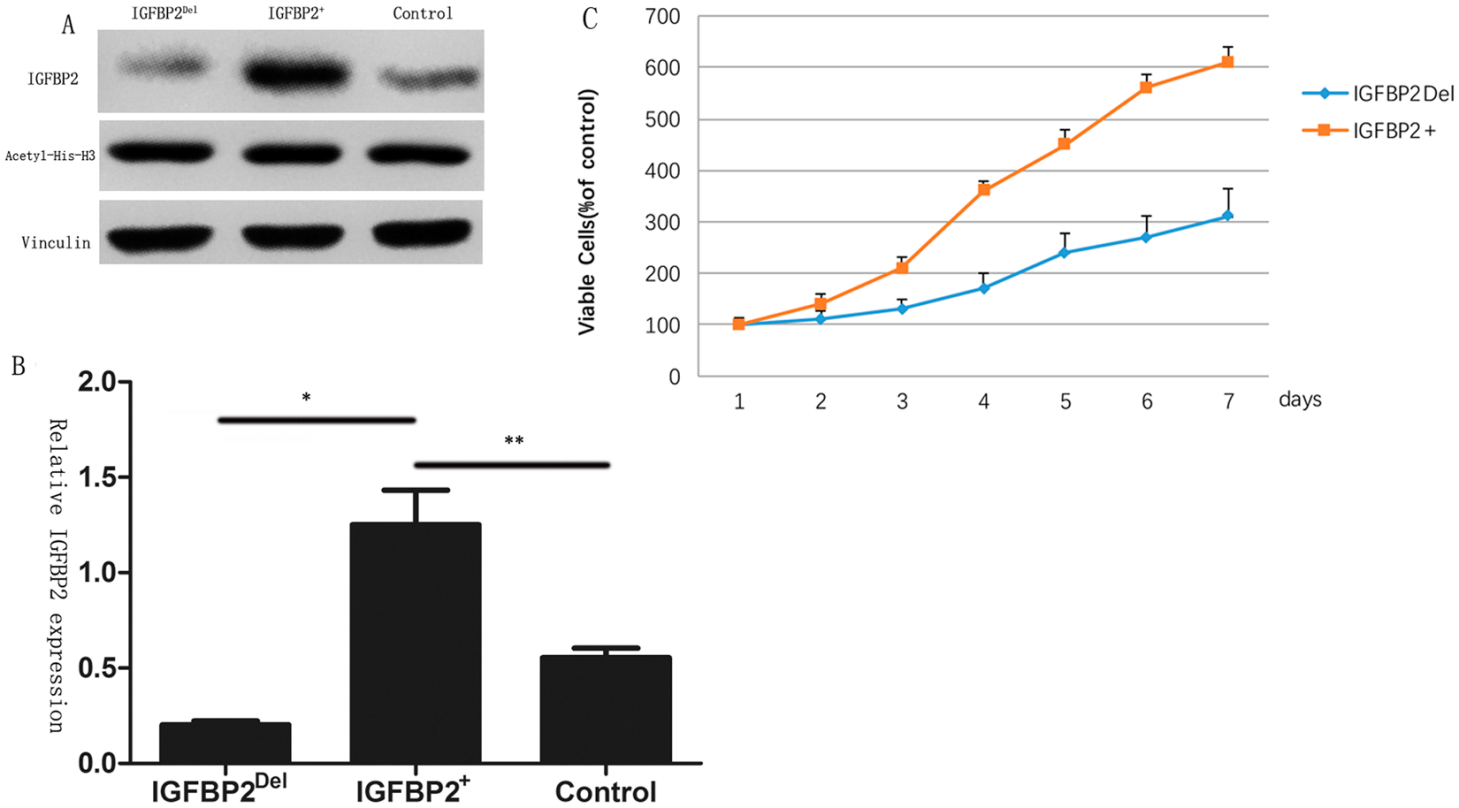
(**A** and **B**) IGFBP2 expression is higher significantly in A549-IGFBP2 cells than control and IGFBP2- knock-down cell lines; (**C**) A549-IGFBP2 cells began to show significant growth advantages compared with knock-lentivirus-infected cells.

Firstly, we detected the expression of IGFBP2 and Acetyl-His-H3 after treating with lentivirus in A549 cells, Western blot (Fig. 3A) and qPCR (Fig. 3B) have verified the constant Acetyl-His-H3 and the expression of IGFBP2 in these transfected cell lines. A549-IGFBP2 cells began to show significant growth advantages after 4 days compared with knock-lentivirus-infected cells (Fig. 3C).

**Figure 4:**
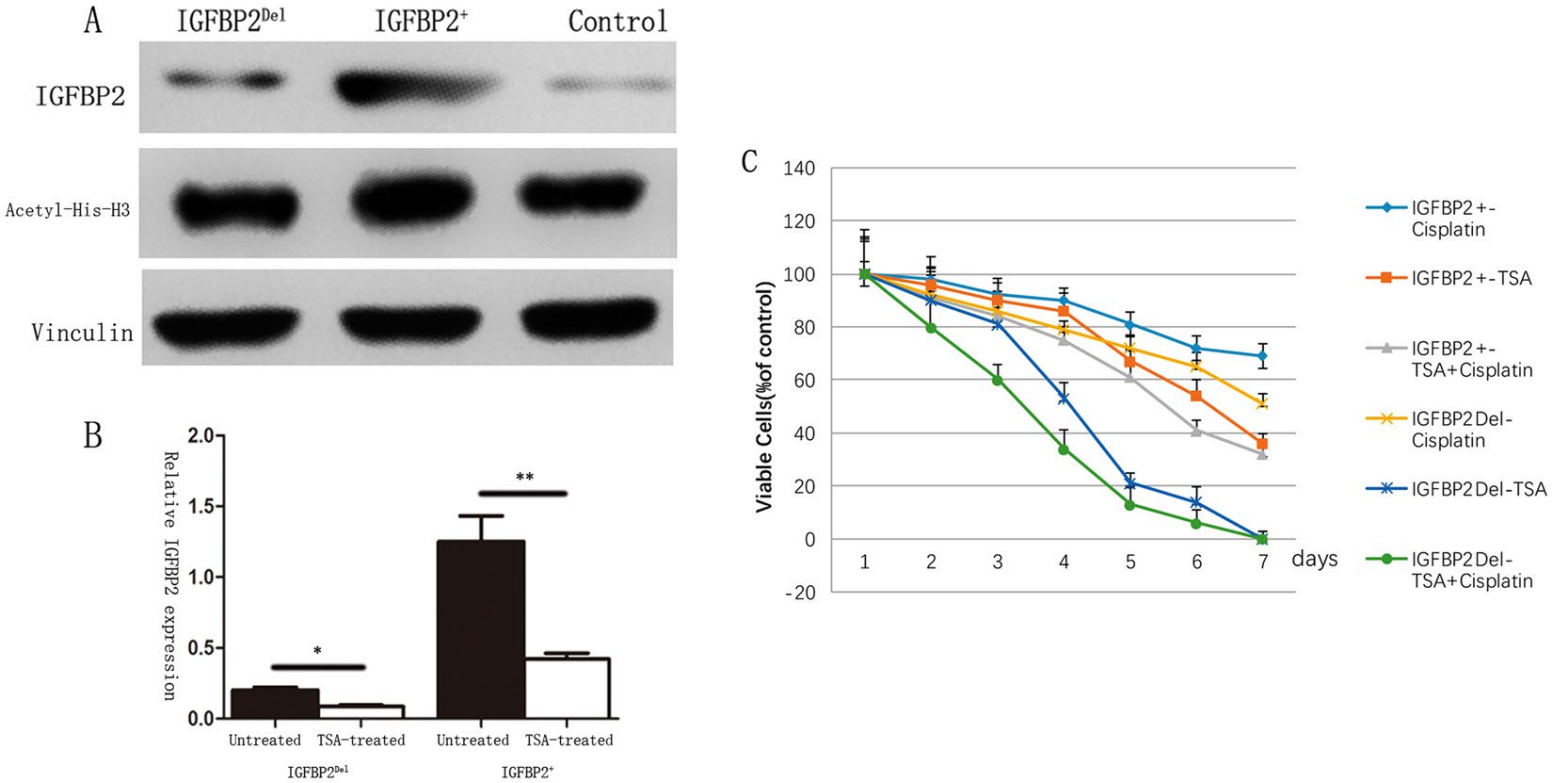
(**A**,**B**) Treatment of A549-IGFBP2 cells with TSA reduced expression levels more significant than IGFBP2- knock-down cells. (**D**) The different effects of TSA, cisplatin and TSA combined with cisplatin on A549-IGFBP2 cells and IGFBP2-knock-down cells suggesting that TSA has re-sensitizing effect on cisplatin-resistant cells.

Next, we detected the expression of IGFBP2 and Acetyl-His-H3 after treating with TSA; and found that treatment of A549-IGFBP2 cells with TSA reduced IGFBP2 and Acetyl-His-H3 expression levels more significant than IGFBP2- knock-down cells (Fig. 4A and B), suggesting that IGFBP2 is the target of TSA which acts on Acetyl-Histone-H3. Furthermore, we compared the different effects of TSA, cisplatin and TSA combined with cisplatin on A549-IGFBP2 cells and IGFBP2- knock-down cells, as a result, TSA has re-sensitizing effect on cisplatin-resistant cells (Fig. 4C).

**Figure 5:**
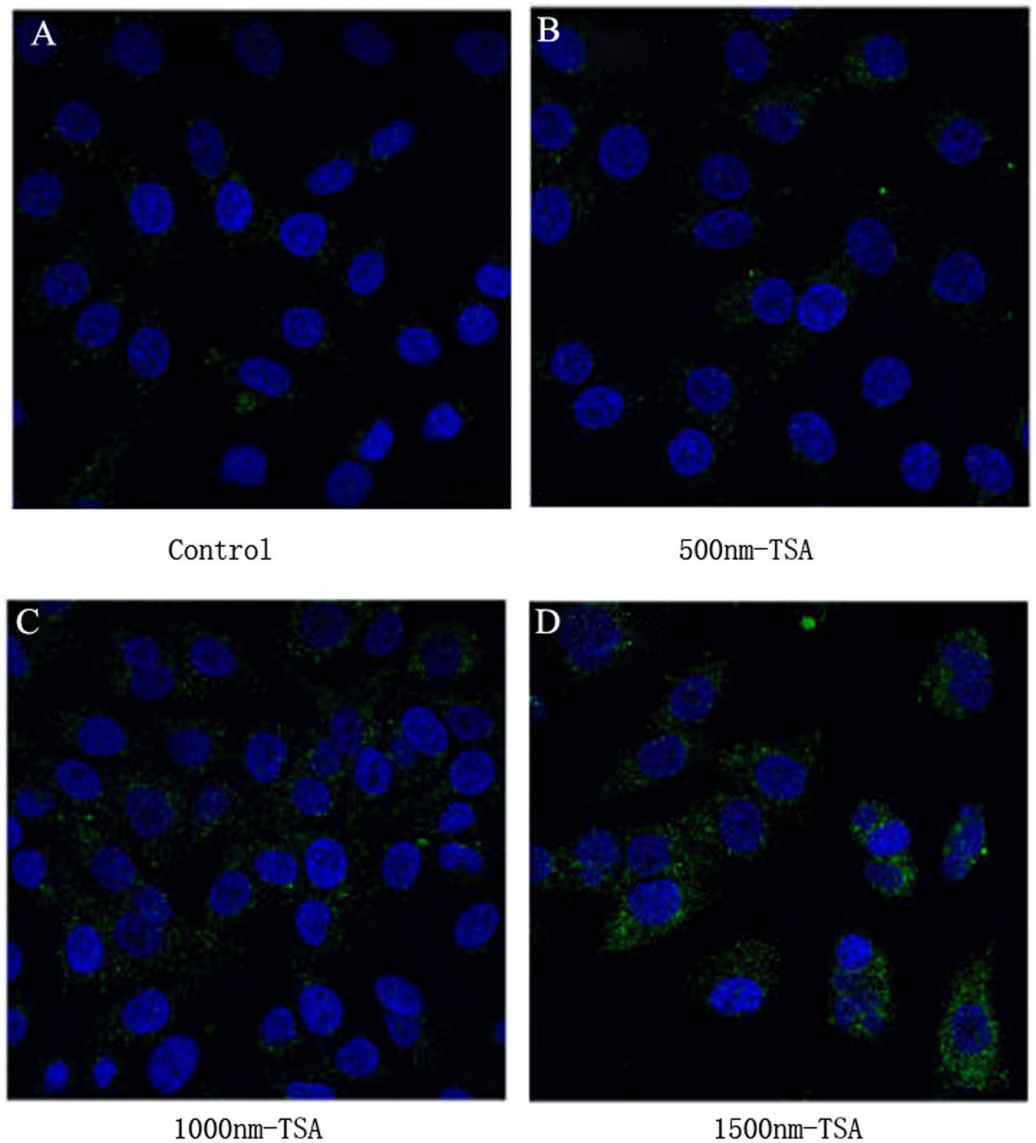
Autophagy increased in dose-dependent manner in A549-IGFBP2 cells after TSA treatment. (**A**) Control; (**B**) 500 nm TSA treatment; (**C**) 1000 nm TSA treatment; (**D**) 1500 nm TSA treatment.

In order to investigate the mechanism of re-sensitizing on cisplatin-resistant cells, we examine the autophagy in A549-IGFBP2 cells. As LC3 could be the marker of autophagy, we detected autophagy using immunofluorescence and quantified the ratio of autophagy cells. And found that autophagy increased in dose-dependent manner (P < 0.05), Fig. 5A showed the results without TSA treatment and detected almost no autophagy; Fig. 5B showed the results with 500 nm TSA treatment and detected about 10% of cells have autophagy; Fig. 5C showed the results with 1000 nm TSA treatment detected about 32% of cells have autophagy; Fig. 5D showed the results with 1500 nm TSA treatment detected about 71% of cells have autophagy; Student *t-*test showed that autophagic intensity improved with the drug concentration increase, so it suggests that TSA reverse the chemoresistance through enhancing autophagy (Fig. 5).

**Figure 6:**
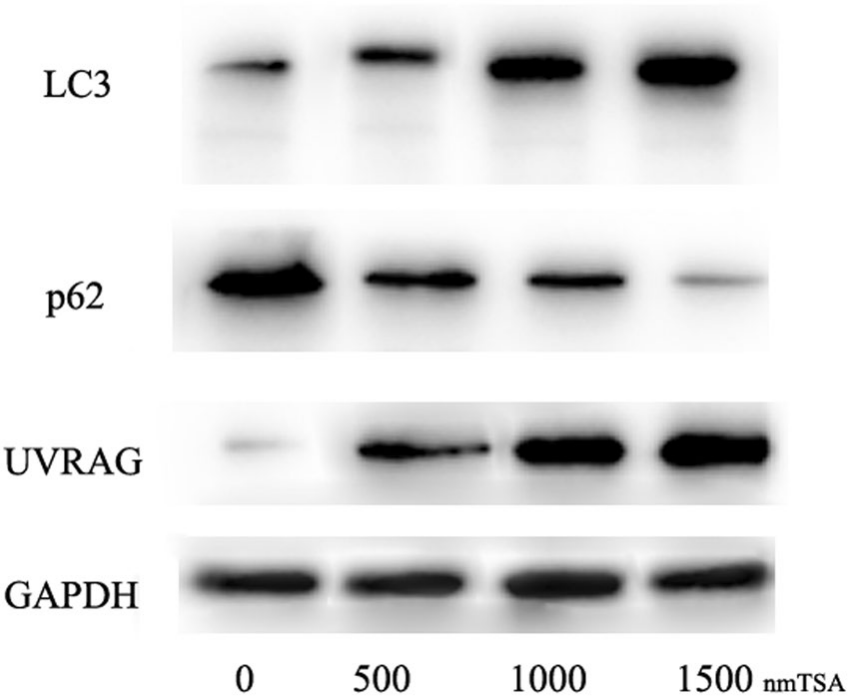
Western blot shows the expression of different autophagy markers; LC3 and the initiator proteins UVRAG increased in dose-dependent manner, p62 decreased significantly in dose-dependent manner.

To further validate the autophagic flux, different autophagy markers were analyzed by Western blot. As a result, LC3 and the initiator proteins UVRAG increased in dose-dependent manner(P < 0.001), whereas, the degradation protein of p62, an adaptor protein which serves as an autophagy receptor targeting ubiquitin proteins to autophagosomes for degradation; decreased significantly(P < 0.001), indicating an enhanced autophagic flux (Fig. 6).

### Association of IGFBP2 with clinical outcome

**Figure 7:**
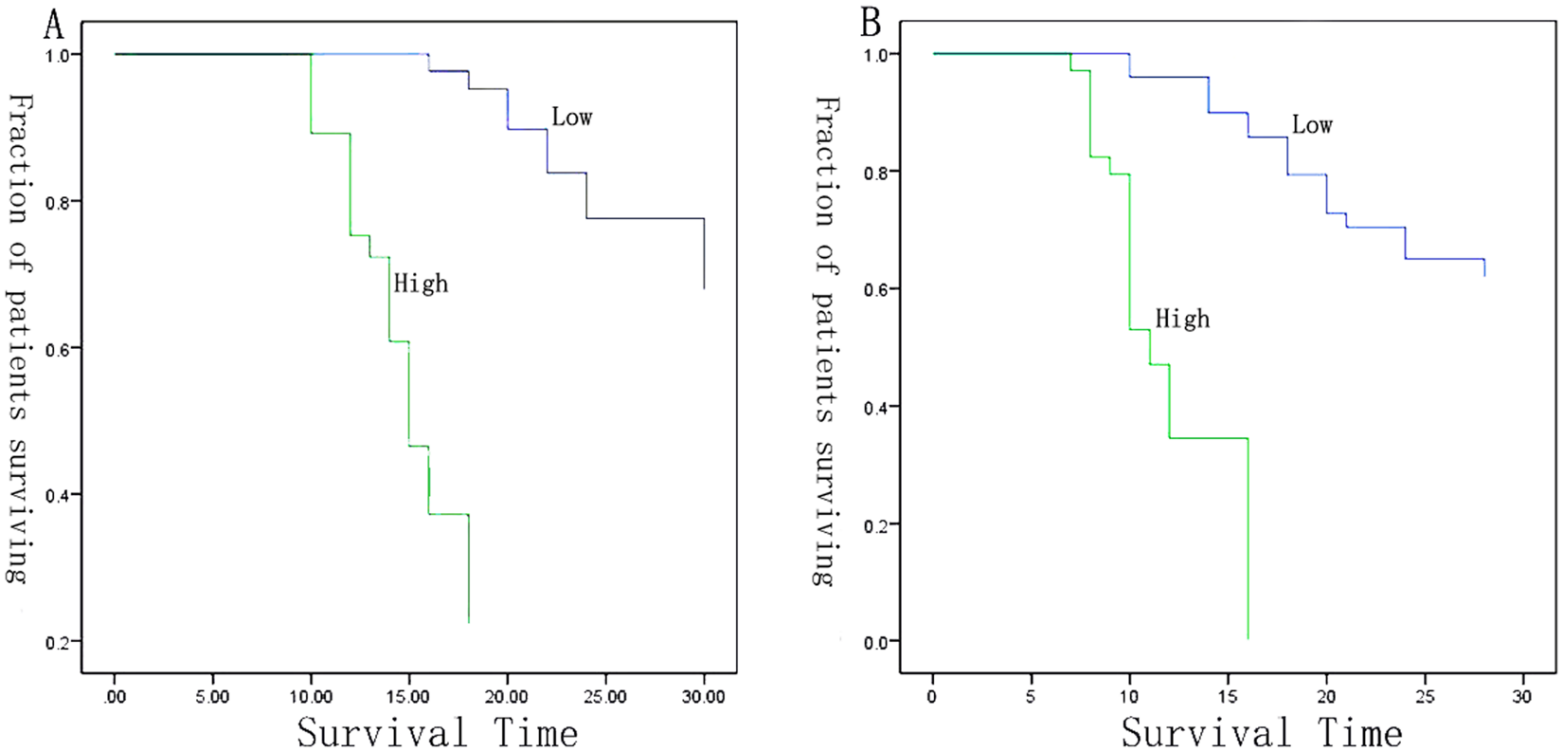
The survival curve of training set and validation set in multivariate analysis, patients with higher IGFBP2 was significantly shorter than that with lower IGFBP2 expression. (**A**) Training set; (**B**) Validation set.

Univariate analysis showed that the survival is significantly correlated with disease stages, differentiation, and IGFBP2 level (Fig. 7A). A multivariate analysis showed that lung cancer patients with higher IGFBP2 had a poor survival outcome (HR = 8.22; 95%CI 1.78–37.92, P = 0.007) after adjustment for histopathology, surgery, pathology, age, smoking history and stage (Fig. 7B). The median survival time for patients with higher IGFBP2 was significantly shorter than that for patients with lower IGFBP2 expression level (14.5 months vs 25.9 months, P < 0001).

## Discussion

The identification of prognostic factors and aberrant signaling pathways is important for new molecular targeted therapy and might improve risk-adapted strategies for lung cancer patients. Here, we have demonstrated that aberrant expression of IGFBP2 may be one novel biomarker for chemoresistance and poor prognosis in NSCLC patients.

Jianhua Zhou *et al*.^7^ have reported that expression of IGFBP2 was observed in both squamous cell lung cancers and adenocarcinomas in 110 NSCLC patients, whereas the positive rate in adenocarcinoma was significantly higher than that in squamous cell carcinoma. Furthermore, IGFBP2 expression was found to be associated with metastasis and poor overall survival in lung cancer. In this study we concentrated on the value of serological IGFBP2 to identify different tumor characteristics in NSCLC patients and the reversal effect of trichostatin A (TSA) on chemoresistance induced by high IGFBP2 expression. As a result, serum IGFBP2 levels are associated with tumor stage and histological differentiation, which is consistent with He Y *et al*. have reported^14^. In addition, for those patients with highest preoperative IGFBP2 levels, it is more likely to recur and poorer overall survival; and TSA could reverse the chemoresistance induced by high IGFBP2 expression through enhancing autophagy. Based on above research, we concluded that IGFBP2 may identify those patients who need further individualized treatment.

The association of increased blood IGFBP2 with shorter overall survival in lung cancer patients^15^; suggests that IGFBP2 may contribute to cancer progression. However, where the elevated IGFBP2 comes from and how to promote tumor progression? Jianhua Zhou *et al*. revealed that the majority of IGFBP2 localized in the cytoplasm. Presently we found that the mean IGFBP2 in the circulation was significantly higher in lung cancer patients than that in healthy and benign participants; and increased with disease progressed to advanced stage. Therefore, we hypothesized that it is possible that IGFBP2 comes from the tumor, and is one of the autocrined hormones. Likely, Ranke MB *et al*. have revealed that IGFBP2 plays an important role in signal transduction and metabolic homeostasis in tumors, including exert an autocrine effect on cancer cells and paracrine effects on endothelial cells and other mesenchymal cells^16^; Additionally, IGFBP2/integrin/ILK/NF-κB network was one key player in glioma progression^17^, intracellular IGFBP2 could regulate caspase-3 expression and contribute to the inhibitory effect on apoptosis^18^. Furthermore, IGFBP2 could be translocated into nucleus and activate other pro-tumorigenic molecules, especially vascular endothelial growth factor (VEGF) which will promote angiogenesis and thus facilitate metastasis^19^. There are studies demonstrated that IGFBP2 interplays with tumor microenvironment; such as IGFBP2 binding to extracellular matrix plays a vital role in proliferation, migration and invasion of neuroblastoma, and acts as a regulator of cancer-mediated endothelial recruitment and angiogenesis in NSCLC^20^. Combined with our results, we concluded as follows: (1) It is likely that IGFBP2 is one of autocrined hormones to activate other signal transduction, (2) IGFBP2 may regulate epithelial-mesenchymal transition and VEGF to promote tumor formation and progression; (3) IGFBP2 plays an important role in tumor migration and invasion. Therefore, we inferred that IGFBP2 may exert in early micro-infiltration process of lung adenocarcinoma. Next, we are ready to collect early lung adenocarcinoma tissues and detect IGFBP2 expression, and look forward to provide some evidence for the progression of early NSCLC.

The development of resistance to chemotherapy remains a challenging issue in the management of lung cancer. Therefore, there is a great need for better understanding of the progression and identification of new therapeutic targets that may improve effective treatment^21,22^. One of the established factors is the loss of the tumor suppressor PTEN. A screen for markers of PTEN identified IGFBP2 as being the most significantly associated one^23^. Recently, studies have shown that IGFBP2 expression may be regulated through the PI3K/Akt/mammalian target of rapamycin signaling pathway^24^. Using the MCF-7 breast cancer cell line, Martin *et al*. showed that inhibition of the PI3K signaling pathway using LY294002 or the mammalian target of rapamycin inhibitor rapamycin, IGFBP2 was markedly reduced; conversely, activation of PI3K signaling increased IGFBP2 levels^25^. And IGFBP2 may regulate PTEN, a key negative regulator in PI3K signaling pathway in MCF-7 cells. However, Levitt *et al*. showed that overexpression of PTEN in the PTEN-null glioma cell line U241 reduced IGFBP2 expression at the mRNA and protein levels^26^. Combined above results we concluded that (1) IGFBP2 is the downstream target of the PI3K signaling; (2) IGFBP2 induces chemoresistance through regulating PTEN. In this study, we discovered that IGFBP2 may predict chemotherapeutic resistance, and Bingliang Fang *et al*. reported that the IGFBP/FAK pathway is associated with dasatinib resistance in lung cancer^22^. In addition, we also revealed that TSA has re-sensitizing effect on cisplatin-resistant cells and reverse the chemoresistance through enhancing autophagy. Therefore, the present study is important in guiding clinical individualized therapies.

## Materials and Methods

### Cell culture and regents

A549 cells (human lung cancer cells) and A549/DDP cells (DDP-resistant human lung cancer cells) were purchased from MeiXuan Biological Science and Technology, Inc. (Shanghai, China), and cultured at 37 °C in F12K medium supplemented with 100 ml/l fetal bovine serum (FBS), 100 kU/l penicillin, and 100 mg/l chloramphenicol in a cell incubator with 5% CO_2_. Trichostatin A (TSA) and cisplatin were purchased from Sigma (St. Louis, MO).

### Patients

The study comprises training and validation subsets. The training set was a retrospective study with 81 NSCLC patients who were enrolled between September 2011 and October 2012, 51 of whom underwent curative surgery at HuaDong Hospital, FuDan University; 30 with unresectable disease or distant metastasis. Simultaneously, 36 healthy or benign pulmonary lesion participants were enrolled to compare with training set. The participants’ characteristics are listed in Table 1. The validation set was prospective cohort study to further verify the ability of IGFBP2 in prognosis and chemoresistance. 84 patients were enrolled between April 2012 and June 2013, 56 of whom underwent potentially curative resection, 28 with unresectable disease or distant metastasis.

For patients who not receiving curative surgery, they will be administered with a minimum four cycles of platinum-based chemotherapy. The main eligibility criteria were: cytologically or histologically proven (according to The World Health Organization) NSCLC, unresectable disease, measurable disease, Eastern Cooperative Oncology Group (ECOG) PS 0–2, and no associated serious co-morbidities.

For NSCLC patients who were cytologically or histologically proven adenocarcinoma, the EGFR gene mutation analysis is needed commonly^27^, targeted therapy will be administered for the patients whose gene analysis results were positive except for clinical stage IA. However, the conventional platinum-based chemotherapy will be administered for the patients whose results were negative except for clinical stage IA.

Initial variables investigated including IGFBP2, age, gender, clinical stage, ECOG PS, tumor histology, smoking history, and CT scan of the chest and the upper part of the abdomen. A bone scintigram and brain CT or magnetic resonance imaging (MRI) were also used for patients with distant metastasis. Objective response was assessed according to the RECIST response criteria: complete response (CR) was complete disappearance of all objective evidence of disease for at least 4 weeks; partial response (PR) was ≥30% reduction in size (products of the longest perpendicular diameter) of measurable lesions without any new lesion for at least 4 weeks; progressive disease (PD) was ≥20% increase in size of known lesions or appearance of new lesions; stable disease (SD) was all other situations. The responders included CR and PR patients; correspondingly, non-responders contained SD and PD patients.

Surveillance proceeded until September 2016 with a median follow-up period of 36 months. Clinical information was obtained by all participants. Informed consent was obtained from all the patients. The ethical committee of HuaDong Hospital Affiliated to FuDan University approved the research protocol. We confirm that all methods were performed in accordance with the relevant guidelines and regulations.

#### Serum specimens

Serum samples were collected from NSCLC patients (median age, 64 years; range, 41–89) before their surgery. Postoperative samples were obtained one week after surgery. Serum samples were additionally obtained in those patients requiring chemotherapy just before each cycle of treatment. Additional serum was collected at 3, 6 months’ follow-up in patients remaining in remission, or at relapse in patients with recurrent disease. Pre- and 1-week postoperative serum was collected in patients with benign pulmonary lesion undergoing surgery (n = 12) during the study, and healthy participants (n = 24, only at the beginning of study).

### Real-time RT-PCR

5 ml of blood sample was obtained and total RNA was extracted. Schizolysised and suppressed the degradation of RNA with guanidine salt and absorbed the production with the specific absorbent, then RNA of high purity could be got after washing and dialysing the production with the detergent and dialysate. In this experiment, separated the total RNA from HL-60 cell strain through the method mentioned above, and amplified Human TFR (Transferrin Receptor) gene by RT-PCR. Primers for GAPDH and IGFBP2 were as follows:

IGFBP2 probe 5′-FAMCCTGCCAGGACTCCCTGCCAAC-TAMRA,

IGFBP2 forward 5′-CATCACCTTGGCCTGGAG,

IGFBP2 reverse 5′-GGATGTGCAGGGAGTAGAGG.

GAPDH probe 5′-HEXATGCCATCACTGCCACCCAGAAGAC-BHQ1,

GAPDH forward 5′-GGTATCGTGGAAGGACTCATGAC,

GAPDH reverse 5′-ATGCCAGTGAGCTTCCCGTTCAG;

IGFBP2 expression was calculated with the mean of the cycle number difference of the two replicates. The amount of mRNA in each sample was then automatically measured by reference to the standard curve constructed each time on the LightCycler software.

### Lentiviral infection of IGFBP2 into A549 cells

The full length cDNA for human IGFBP-2 was subcloned into the lentiviral vector pHR’-CMV-EGFP at the BamHI and XhoI sites. Two vectors were created for study: pHR’-CMV-IGFBP-2 and pHR’-CMV (empty vector). Clone identity was verified using restriction digest analysis and plasmid DNA sequencing. Infectious lentivirus was generated by cotransfection of 1.5 * 106 293 T cells with target plasmids with pCMVDR8.2 (carries sequence necessary for viral assembly of lentivirus) and pMD.G, which expresses the vesicular stomatitis virus envelop glycoprotein G pseudotype as described previously^28^. The 293-T cells were transfected for 12 to 15 h, after which fresh medium was added for 24 h. After this, the virus-containing medium was collected and passed through a 0.45 Am filter. Early-passage A549 cells were plated on 10-cm plates, and competent retrovirus was added to 30 to 40 multiplicities of infection. The medium was changed after incubation for 16 h. The cells were passaged and harvested for UV microscopy to verify green fluorescent protein expression. Cell lysate were collected to ensure expression of IGFBP-2.

### Microscopy

Cells were plated at low confluence in 6-well plates (50,000 cells/well). Onday2, cells were exposed to serum starvation (0% FBS), normal medium (10% FBS), or chloroquine (50 mmol/L) for 24 hours. Medium was removed, cells were washed with PBS and treated with 4% paraformaldehyde/PBS for 20 minutes at room temperature, washed, and then permeabilized with 0.1% Triton X-100 for 10 minutes. Cells were then blocked with 5% normal goatserum (Cell Signaling Technology) containing 0.3% Triton X-100 in PBS for 60 minutes. Diluted primary antibody, anti-mouse LC3 A/B (Cell Signaling Technology), was applied in blocking buffer overnight at 4 °C. Alexa Fluor-555 secondary antibody diluted in 1% normal goat serum in PBS were added for 1 hour at ambient temperature. Cells were fixed using Vectashield hard set mounting medium containing DAPI dye (Vector Laboratories). Images were acquired using confocal microscopy (Olympus FV-1000) and overlaid using ImageJ.

### Western blot analysis

Cells were harvested by lysis in radioimmunoprecipitation assay buffer and protease inhibitors sheared with a 26-gauge needle. Protein (30Ag) was separated by 10% PAGE, transferred to 0.45 Am Immobilon-P Transfer membranes (Millipore), and analyzed by Western blotting with anti-LC3, p62, UVRAG, IGFBP2 antibodies, Acetyl-Histone H3 (Lys9) (C5B11) Rabbit (untreated or TSA-treated (400 nM for 18 hours) using Acetyl-Histone H3 (Lys9) (C5B11) Rabbit mAb) (Cell Signaling Technology). Loading levels were normalized using 1:2,000 antivinculin antibodies (Sigma) and densitometric analysis. To determine the amount of secreted IGFBP-2, conditioned medium obtained from cell lines cultured for 48 h in serum-free DMEM was used to perform Western blot analysis for IGFBP-2 as described above.

### Flow cytometric analysis

A549^Del^, A549^+^, and A549Mock cells were plated in 75 cm^2^ dishes and treated as described above the following day. The cells were trypsinized 2 days after either TSA; Cisplatin or Cisplatin + TSA treatment and analyzed for relative DNA content on a dual laser flow cytometer (Beckman Coulter Epics Elite; Beckman). Each assay was done in triplicate.

### Statistical analysis

The cutoff value was determined by ROC curve. The patient was considered positive whose value was above the cutoff. Survival was analyzed by Kaplan–Meier curves with death and lost as censored. Fisher’s exact test and Student’s t-test were employed. Independent prognostic factors were performed in univariate and multivariate analysis using the Cox hazard model. The data presented in bar graph as mean ± SD; SPSS 21.0 software were applied with P ≤ 0.05 was considered to be significant.
